# Finite Element Analysis of Strengthening Mechanism of Ultrastrong and Tough Cellulosic Materials

**DOI:** 10.3390/polym14214490

**Published:** 2022-10-24

**Authors:** Xiaoshuai Han, Jingwen Wang, Xiaoyi Wang, Wei Tian, Yanyan Dong, Shaohua Jiang

**Affiliations:** 1 Jiangsu Co-Innovation Center of Efficient Processing and Utilization of Forest Resources, International Innovation Center for Forest Chemicals and Materials, College of Materials Science and Engineering, Nanjing Forestry University, Nanjing 210037, China; 2 Institute of Environment and Sustainable Development in Agriculture, Chinese Academy of Agricultural Sciences, Beijing 100081, China

**Keywords:** wood, cellulose nanofibril, hydrogen bonding, strengthening, finite element analysis (FEA)

## Abstract

Superior strong and tough structural materials are highly desirable in engineering applications. However, it remains a big challenge to combine these two mutually exclusive mechanical properties into one body. In the work, an ultrastrong and tough cellulosic material was fabricated by a two-step process of delignification and water molecule-induced hydrogen bonding under compression. The strong and tough cellulosic material showed enhanced tensile strength (352 MPa vs. 56 MPa for natural wood) and toughness (4.1 MJ m^−3^ vs. 0.42 MJ m^−3^ for natural wood). The mechanical behaviors of ultrastrong and tough bulk material in a tensile state were simulated by finite element analysis (FEA) using mechanical parameters measured in the experiment. FEA results showed that the tensile strength and toughness gradually simultaneously improved with the increase in moisture content, demonstrating that water molecules played an active role in fabricating strong and tough materials, by plasticizing and forming hydrogen bonding among cellulose nanofibrils.

## 1. Introduction

High-performance structural materials with high strength and fracture toughness have consistently drawn significant attention and are in great demand in the modern manufacturing industry [1,2,3,4,5,6]. When developing such high-strength and tough structural materials, natural polymers should be preferred due to their renewable and sustainable characteristics [7,8]. Cellulose is the most abundant biopolymer on Earth, found in trees, bamboo, rattan, agricultural crops, and other biomass, even bacteria [9,10,11,12,13,14]. Cellulose possesses attractive intrinsic mechanical properties with tensile strength of about 3.0−4.7 GPa g^−1^ cm^3^ and a theoretical modulus of about 63−125 GPa g^−1^ cm^3^ in its crystal region [15,16], both of which are higher than most metals, alloys, and some ceramics, which makes it an ideal building block for a series of strong and tough materials, such as nanopaper, films, and superstrong wood [17,18,19].

In general, cellulose-derived materials (cellulose nanofibrils or cellulose crystals) could be directly bought from a factory and corresponding structural materials can be fabricated using bottom-up assembly methods such as vacuum filtration, layer-by-layer assembly, or solvent casting [20,21,22,23,24]. However, this bottom-up assembly method has some intrinsic drawbacks including (1) cellulose-derived materials’ isolation is chemical–energy intensive; (2) the assembly process is time-consuming and laborious and complicated; (3) the products are usually on a small scale and hard to transfer to bulk materials. Recently, a top-down recombination method has been adapted to fabricate large-scale cellulosic structural materials due to their natural disadvantages of aligned nanocellulose structure [25,26,27,28,29,30]. During the fabrication process, some kinds of organic (epoxy, phenolic resin, etc.) or inorganic polymers (nanosilica, calcium carbonate, etc.) were combined with a cellulosic framework to improve the physical and mechanical properties of products [31,32,33]. Our group also proposed a simple yet universal top-down method for making strong and tough structural materials by water molecule-induced hydrogen bonding. This method can convert natural wood into strong and tough bulk materials by a three-step process of delignification, drying-induced assembly, and water molecule-induced hydrogen bonding under compression. A tough and strong cellulose nanofiber bulk material was derived, showing simultaneously enhanced tensile strength (352 MPa vs. 56 MPa for natural wood) and toughness (4.1 MJ m^−3^ vs. 0.42 MJ m^−3^ for natural wood) [34]. Although we analyzed the plasticizing and strengthening mechanism of water molecules for cellulose nanofibrils, this explanation can only be indirectly characterized by macroscopic physical and mechanical data. Some visible means of characterization is necessary for identifying the water molecule-induced strengthening mechanism between cellulose nanofibrils. Finite element analysis (FEA) has been commonly used in engineering fields by virtue of developing technologies [35,36]. Meanwhile, FEA has also been confirmed as an effective method commonly used in wood engineering [37,38].

In this study, a methodological means was proposed: the water molecule-induced hydrogen bonding effect between cellulose nanofibrils was investigated numerically by first using FEA. The relationship between hydrogen bonding and tensile properties was analyzed by comparing FEA simulation and experimental data.

## 2. Materials and Methods

### 2.1. Materials and Chemicals

Basswood with dimensions of 50 mm (L) × 50 mm (T) × 10 mm (R) was used in this study. Sodium chlorite (NaClO_2_, 80%), sulfuric acid (H_2_SO_4_, 72%), acetic acid (HAc, 99.7%), potassium sulfate (K_2_SO_4_), acetone (99.8%), and hexane (99.8%) were purchased from Fisher Scientific (Thermo Fisher Scientific Inc., Shanghai, China). All chemicals were used as received without further purification. Deionized (DI) water was used for sample preparation.

### 2.2. Fabrication of Ultrastrong and Tough Cellulose Nanofibril Bulk Material

Basswood was chemically treated to remove most lignin and partial hemicellulose and then dried using a solvent exchange drying method, followed by compression to the final ultrastrong and tough cellulose nanofibril bulk material according to a previous report [34]. In this study, NW stands for natural basswood, DW stands for delignified wood, DW_SD_ stands for delignified wood by solvent exchange drying, CDW_SD_ stands for compressed delignified wood by solvent exchange drying, followed by drying at 105 °C; CDW_SD0_ stands for compressed delignified wood under 0% moisture content, followed by drying at 105 °C; CDW_SD9_ stands for compressed delignified wood under 9% moisture content, followed by drying at 105 °C; CDW_SD18_ stands for compressed delignified wood under 18% moisture content, followed by drying at 105 °C.

### 2.3. Characterization

The chemical compositions of both natural wood (NW) and delignified wood (DW) were qualitatively analyzed by a Nicolet 6700 infrared spectrophotometer (IR, Thermo Scientific, Waltham, MA, USA) equipped with an ATR accessory. The lignin contents (Klason lignin) of NW and DW were determined according to a standard TAPPI T 222 om-2 method [39]. The morphologies of NW, DW, compressed delignified wood by solvent exchange drying compressed under 0% moisture content (CDW_SD0_), 9% moisture content (CDW_SD9_), and 18 moisture content (CDW_SD18_) were characterized using a Phenom XL G2 Desktop scanning electron microscope. The mechanical properties of the samples were measured using an Academic User Instron 5969 Uniaxial Materials Testing System (Instron 5969, Norwood, MA, USA). At least ten specimens were tested for all samples and the averages and standard deviations were presented.

The densities of NW, DW_SD_, CDW_SD0_, CDW_SD9_, and CDW_SD18_ were determined from the ratios of mass to volume. The porosities of the above samples were calculated as follows:

$$\mathrm{Porosity}\;\left(\%\right)=\left(1-\frac{\rho_{a}}{\rho_{b}}\right)\times 100\%$$

where $\rho_{a}$ is the density of the cellulose nanofiber bulk material and $\rho_{b}$ is the density of the delignified wood, taken as 1.5 g∙cm^−3^.

### 2.4. Numerical Modeling of Ultrastrong and Tough Bulk Material

The fabricated ultrastrong and tough bulk material is simulated for validation of the model for strength, stiffness, and failure mechanisms by macro and micro finite element analysis (FEA). The macro FEA of the tension test based on a 3D solid model was performed. The micro FEA construction of tension test included two steps: (1) the length of cellulose nanofibril was assumed to be 7.5 μm and the transverse section’s side length was 3.5 nm. The vertical distances between cellulose nanofibrils were 0.29 nm (0% moisture content), 0.17 nm (9% moisture content), and 0.13 nm (18% moisture content), respectively. The distance along the cellulose nanofibrils’ ends was assumed to be 0.5 nm. After that, finite element software ABAQUS (ABAQUS version 2014) was used to capture representative bulk unit cells consisting of wood cellulose nanofibrils and mesenchyme. Figure S1 (Supporting Information) shows representative bulk unit cell models of CDW_SD0_, CDW_SD9_, and CDW_SD18_; (2) determination of the conditions for FEA. Based on the representative bulk unit cell model above, the conditions of micro FEA were determined as follows: the fracture strength and Young’s modulus of microfibrils were set as 2.3 GPa and 129 GPa, respectively [10,40]. The strength of nanofibril mesenchyme was unknown and it represented the bonding force between cellulose nanofibrils under different moisture contents. The mechanical properties of mesenchyme were characterized through simulating and comparing the experimental tensile strength and fracture strain data, which revealed bonding force between cellulose nanofibrils of CDW_SD0_, CDW_SD9_, and CDW_SD18_.

## 3. Results

**Scheme 1:**
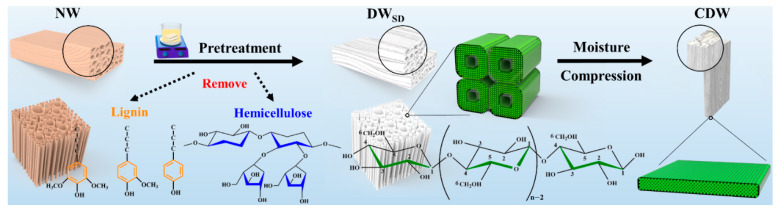
Schematic illustration of the top-down fabrication of ultrastrong and tough cellulose nanofibril bulk material with delignification, solvent exchange drying, moisture absorption, and compression process.

Scheme 1 shows our top-down approach for fabricating ultrastrong and tough cellulose nanofibril bulk material with the aid of water molecule-induced hydrogen bonding. In detail, this material was fabricated following a three-step process: (1) delignification to remove hydrophobic lignin and ionic interacted hemicellulose, which exposed well-aligned cellulose nanofibrils and enhanced hydrogen bonding ability; (2) a solvent exchange drying method was employed to remove water and maintain the original wooden framework facilitating water molecule adsorption; (3) rehydration of solvent exchange dried delignified wood to different moisture content levels, followed by mechanical compression to introduce hydrogen bonding for enhanced mechanical performance.

**Figure 1:**
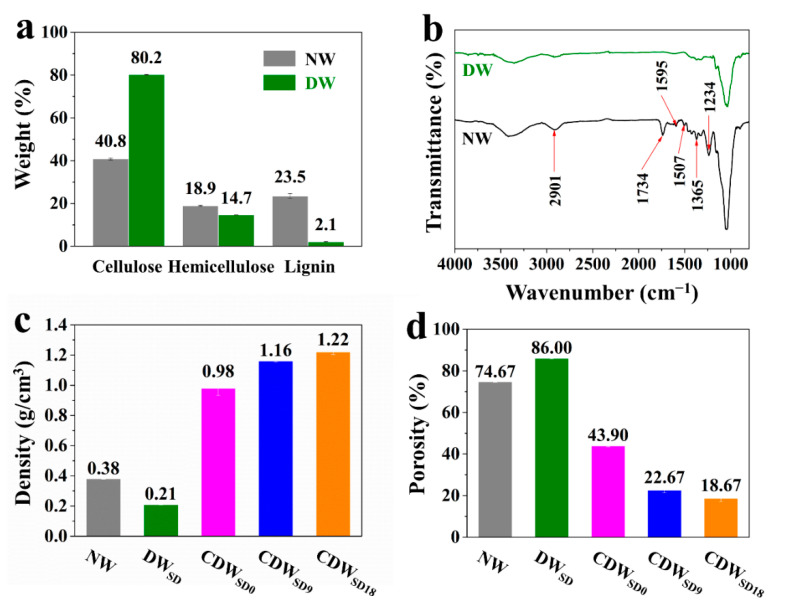
(**a**) The composition of cellulose, hemicellulose, and lignin in natural wood (NW) and delignified wood (DW). (**b**) FTIR spectra of NW and DW. (**c**) Density and (**d**) porosity of NW, delignified wood by solvent exchange drying (DW_SD_), compressed delignified wood by solvent exchange drying compressed under 0% moisture content (CDW_SD0_), 9% moisture content (CDW_SD9_), and 18 moisture content (CDW_SD18_).

In order to investigate the water molecule-induced hydrogen bonding effect on inter-cellulose nanofibrils, the natural basswood was subjected to sodium chlorite (NaClO_2_) and sodium hydroxide (NaOH) treatment to remove lignin and hemicellulose. The result showed that the DW sample was composed of 80.2% cellulose, 14.7% hemicellulose, and 2.1% lignin, indicating 22% hemicellulose and 91% lignin removal (Figure 1a). The delignification result was further validated by FTIR spectroscopy (Figure 1b). There are typical hemicellulose absorption peaks at 1734 cm^−1^ (unconjugated carbonyl C=O) and lignin absorption peaks at 1507 cm^−1^ and 1595 cm^−1^ (C=H stretching of the aromatic rings), 1365 cm^−1^ (symmetric C-H bending from methoxyl group), and 1234 cm^−1^ (C-O stretching of the aromatic rings) in natural basswood. However, the above absorption peaks disappeared in DW after NaClO_2_ and NaOH treatment. As the hemicellulose and lignin were removed, the DW_SD_ had lower density (0.21 g cm^−3^) and higher porosity (86.00%) compared to natural basswood. In order to validate the water molecule-induced hydrogen bonding strengthening effect, the DW_SD_ absorbed different water vapor contents (0% moisture content, 9% moisture content, and 18% moisture content) and was then compressed and dried. The result presented that the density of CDW_SD_ increased and the porosity decreased with increasing moisture content, indicating a positive effect of water molecules for improving the densification degree of the cellulose nanofibrils within DW_SD_ (Figure 1c,d).

**Figure 2:**
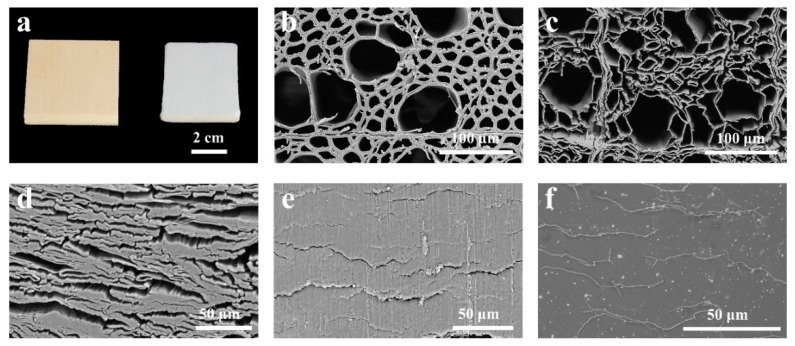
(**a**) Photographs of NW and DWSD. SEM cross-section images of (**b**) NW, (**c**) DW_SD_, (**d**) CDW_SD0_, (**e**) CDW_SD9_, and (**f**) CDW_SD18_.

The morphologies of NW, DW_SD_, CDW_SD0_, CDW_SD9_, and CDW_SD18_ are presented in Figure 2 and Figure S2 (Supporting Information). Compared with NW, the volume of DW_SD_ was slightly reduced (Figure 2a), indicating that the solvent exchange drying technique did not destroy the microstructure of NW. The SEM cross-section images of NW and DW_SD_ (Figure 2b,c) showed the separated individual wood cells, showing non-polar hexane restricted the association between wood fibers. In addition, the wood cell walls of DW_SD_ was thinner than that of NW because of hemicellulose and lignin removal. As we all know, DW is a hygroscopic material, which tends to bind water molecules through hydrogen bonding via its surface hydroxyl groups. In order to investigate the effect of bound water on the densification and mechanical properties of the delignified wood, the DW_SD_ absorbed a given mass of moisture (0%, 9%, and 18%) in a desiccator containing K_2_SO_4_ solution, which can provide 97.6 ± 0.5% relative humidity (RH) at 20 °C. The DW_SD_ having different moisture contents were then compressed and dried. At 0% moisture content, the DW_SD_ could not be compressed to a dense material and the CDW_SD0_ experienced springback, indicating cellulose nanofibrils could not tightly link together. Therefore, there are a lot of big gaps caused by fewer bindings among wood cells (Figure 2d). When increasing moisture content on the surface of cellulose nanofibers, the CDW_SD_ showed more dense and compact cross-section microstructure, which is because water molecules can be plasticizers, increasing the flexibility and softness of cellulose nanofibers and causing wood cell walls to tightly intertwine and densely pack together under compression. Additionally, more bound water molecules on the surface of cellulose nanofibers increased the hydrogen bonding capacity, creating more strong interactions between cellulose nanofibrils. Thus, these big gaps disappeared and there were fewer microcracks in the CDW_SD9_ (Figure 2e). For CDW_SD18_, the cellulose nanofibrils inside cell walls had merged together by compression (Figure 2f). The above results were also consistent with the density and porosity analyses as shown in Figure 1c,d.

**Figure 3:**
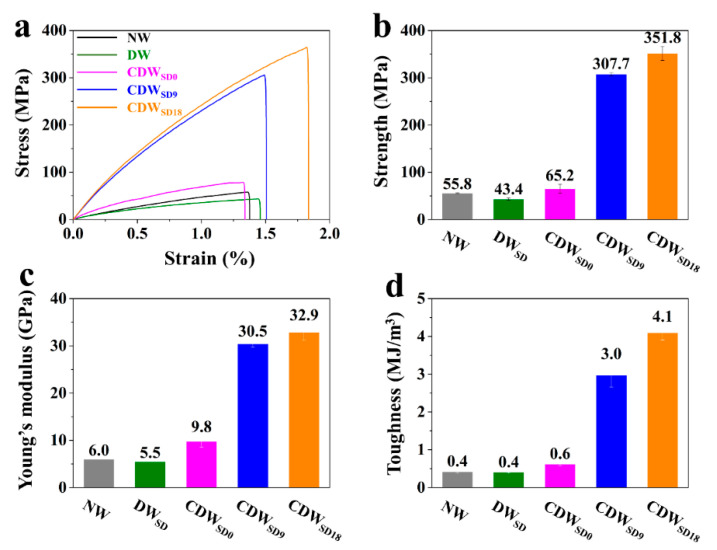
(**a**) Typical tensile stress–strain curves for NW, DW_SD_, CDW_SD0_, CDW_SD9_, and CDW_SD18_. The respective (**b**) tensile strength, (**c**) Young’s modulus, and (**d**) toughness from the tensile stress–strain curves.

Figure 3 demonstrates tensile–strain curves and the corresponding mechanical performance of the NW, DW_SD_, CDW_SD0_, CDW_SD9_, and CDW_SD18_. Due to the loose and separated structure formed during delignification and solvent exchange drying, the DW_SD_ showed low mechanical properties with tensile strength, Young’s modulus, and toughness of 43.4 MPa, 5.5 GPa, and 0.4 MJ m^−3^, respectively, which were lower than those of NW (55.8 MPa, 6.0 GPa, and 0.4 MJ m^−3^). For compressed solvent exchange dried delignified wood, the mechanical properties increased with increasing moisture content prior to compression. The tensile strength (351.8 MPa), Young’s modulus (32.9 GPa), and toughness (4.1 MJ m^−3^) of CDW_SD18_ were more than 5, 3, and 6 times higher than those of CDW_SD0_. As previously discussed, the CDW_SD0_ displayed a springback phenomenon and big gap microstructure due to mutually exclusive hydroxide groups, which could not form enough hydrogen bonds when compressed at 0% moisture content. The mechanical performance change clearly indicated that water molecules played the role of plasticizers and structural molecules between cellulose nanofibrils to enhance the hydrogen bonding interactions of inter-nanofibrils.

**Figure 4:**
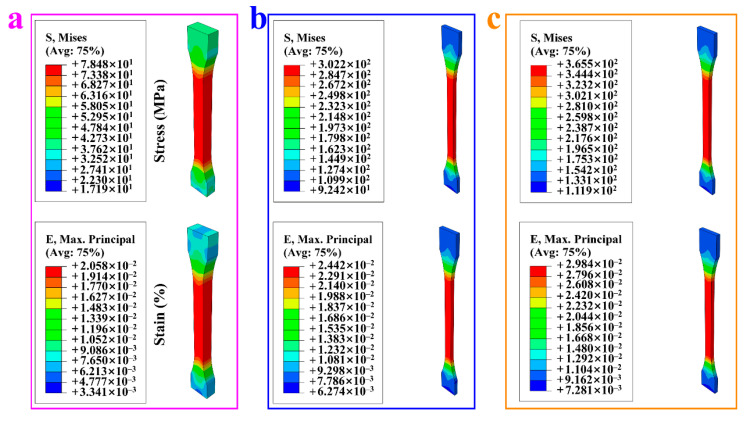
The macro finite element analysis of (**a**) CDW_SD0_, (**b**) CDW_SD9_, and (**c**) CDW_SD18_.

Figure 4 shows the stress distributions of CDW_SD0_ (Figure 4a), CDW_SD9_ (Figure 4b), and CDW_SD18_ (Figure 4c) when subjected to tension load. Meanwhile, Videos S1–S3 (Supporting Information) also present the variations of stress distributions during the loading process. The results of macro FEA indicated that the main loading was located at the middle of the sample. The CDW_SD18_ possessed superior tensile strength (365.5 MPa) compared to CDW_SD9_ (302.2 MPa) and CDW_SD0_ (78.4 MPa), which was also in accordance with the experimental studies.

**Figure 5:**
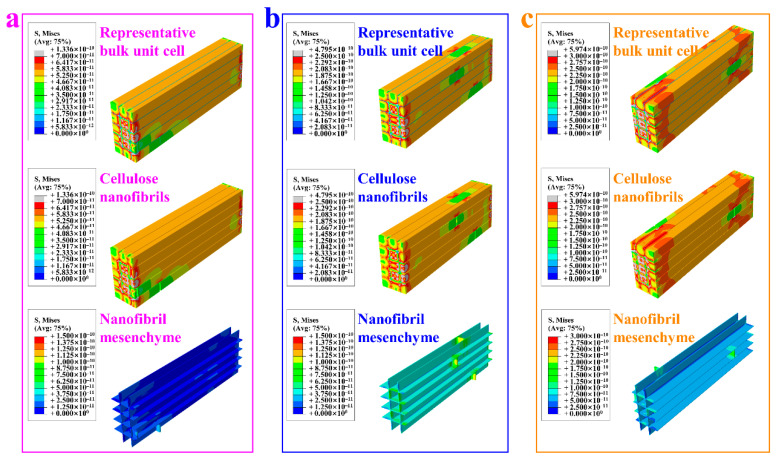
The stress distributions of representative bulk unit cell, cellulose nanofibrils, and nanofibril mesenchyme for (**a**) CDW_SD0_, (**b**) CDW_SD9_, and (**c**) CDW_SD18_ during micro finite element analysis process.

Figure 5 presents the stress distributions of a representative bulk unit cell, cellulose nanofibril, and nanofibril mesenchyme models of CDW_SD0_ (Figure 5a), CDW_SD9_ (Figure 5b), and CDW_SD18_ (Figure 5c) during the micro FEA process. The micro FEA result indicated that the main breaking points belonged to cellulose nanofibril mesenchyme, which was consistent with experimental data. There are some burrs at the breaking point that also proved that the cellulose nanofibril mesenchyme, not cellulose nanofibril, experienced fracture in tensile testing. With the increase in moisture content in compressed samples, the tensile strength was gradually improved, which illustrated that the hydrogen bonding among cellulose nanofibrils was increased from the qualitative perspective. Therefore, the CDW can endure higher stress, which was caused by bigger fracture forces of cellulose nanofibril mesenchyme with the increase in moisture content.

## 4. Conclusions

In this work, we reviewed a strong and tough material and investigated the dynamic tensile deformation behavior of this material by using macro/micro FEA and experimental methods. The deformation modes for compressed delignified wood under different moisture contents subjected to dynamic mechanical stretching were identified. The following conclusions were drawn: (1) the numerical simulation and theoretical analysis results were in accordance with experimental data; (2) FEA was an effective approach to predict the fracture position and force situation for samples under dynamic stress; (3) the results of FEA showed that water molecules can serve as plasticizers and hydrogen-bonding bridges to simultaneously enhance strength and toughness. In conclusion, the proposed macro/micro FEA is capable of being used to evaluate the dynamic mechanical properties and reveal strengthening mechanisms for cellulosic materials, which will contribute to fabricating superior structural composites.

## Data Availability

The data used to support the findings of this study are available from the corresponding author upon request.

## Supplementary Materials

The following supporting information can be downloaded at: https://www.mdpi.com/article/10.3390/polym14214490/s1, Figure S1: Representative bulk unit cell models of (a) CDW_SD0_, (b) CDW_SD9_, and (c) CDW_SD18_; Figure S2: The photograph of DW_SD_ and CDW_SD18_; Video S1: The stress distribution variation of CDW_SD0_; Video S2: The stress distribution variation of CDW_SD9_; Video S3: The stress distribution variation of CDW_SD18_.
