# Supplementary material for: Finite Element Analysis of Strengthening Mechanism of Ultrastrong and Tough Cellulosic Materials
Source: Polymers (Basel). 2022 Oct 24;14(21):4490. doi: 10.3390/polym14214490 (PMC9654337; doi:10.3390/polym14214490)
Supplement: Supplementary file 1 [file polymers-14-04490-s001.zip › polymers-1974934-supplementary.pdf]

Finite element analysis of strengthening mechanism of ultrastrong and tough cellulosic materials

Xiaoshuai Han[1], Jingwen Wang[1], Xiaoyi Wang[1], Wei Tian[1], Yanyan Dong[2,*] and Shaohua Jiang [1,*]

[1] Jiangsu Co-Innovation Center of Efficient Processing and Utilization of Forest Resources, International Innovation Center for Forest Chemicals and Materials, College of Materials Science and Engineering, Nanjing Forestry University, Nanjing, 210037, China.

[2] Institute of Environment and Sustainable Development in Agriculture, Chinese Academy of Agricul-tural Sciences, Beijing 100081, China.

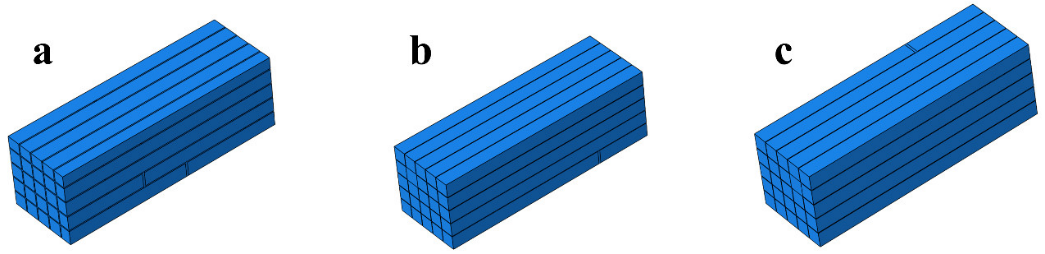

Figure S1. Representative bulk unit cell models of (a) CDW_{SD0}, (b) CDW_{SD9}, and (c) CDW_{SD18}.

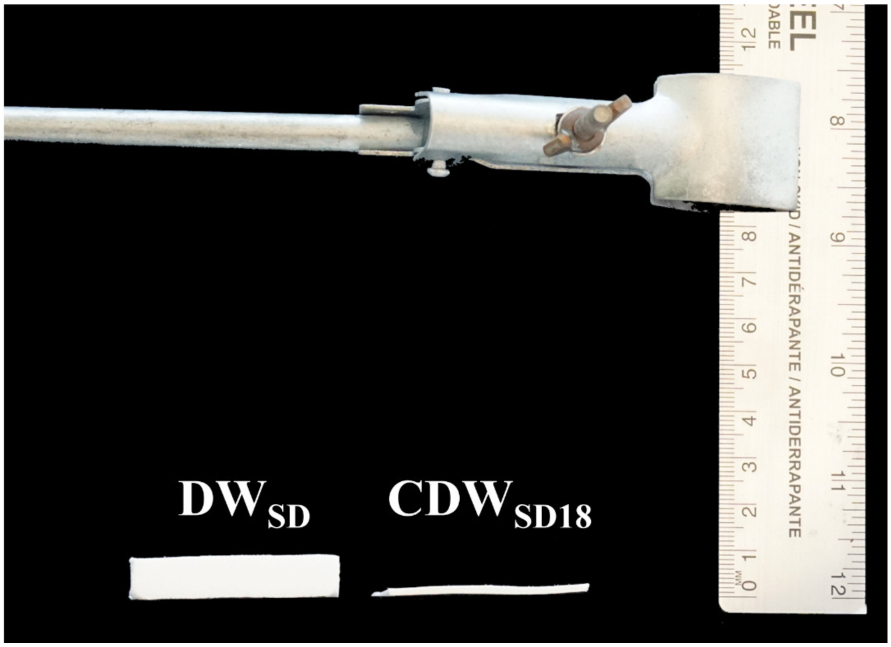

Figure S2. The photograph of DW_{SD} and CDW_{SD18}.
